# Homes for the Dying

**Published:** 1896-09-26

**Authors:** 


### HOMES FOR THE DYING.

### THE TWO EXISTING HOMES.

In THE HOSPITAL for July 18th, we partly dealt with this subject, and in the course of the remarks then made referred to the two Homes for the Dying, viz.: Friedenheim and the Clapham Home. There is also a third home, St. Luke's, Osnaburgh Street, to which reference will be made on another occasion.

The two first-named Homes for the Dying are quite distinct and differ largely in certain respects. In Friedenheim the patients, as the report states, are cared for by a nursing staff, all of whom are professing Christians, and the patients frequently testify to their being veritable "living epistles."

The medical officer, Dr. Lush, in his report, gives an indication of the meaning of the words we have just quoted in the following paragraph:—

> I gladly and thankfully record what I know to be a fact—that the "higher" object which Miss Davidson had in instituting the Home of Peace for the Dying has been amply attained; and as I write the memory of more than one patient comes to me who had no hope and was without God in the world when admitted to Friedenheim, but who, before the end came, found the peace of God to be a transforming reality.

In another part of the report it is stated:—

> The silent teaching of the texts which abound on all the walls has also been found a powerful factor in arousing the attention of the careless, and although there is no forcing of religion on the unwilling it need hardly be said that Miss Davidson, her staff, and her visitors are always ready with a "word in season" as the opportunity presents itself.

These extracts will give the reader a fairer and a better idea of the principles upon which Friedenheim is conducted than anything we could write. Miss Davidson has evidently an earnest desire that everyone should be properly cared for in both a bodily and spiritual sense.

We notice that the rules make no mention of the visits of ministers of religion of all or any denominations, though the report mentions the constant visits of two clergymen, the Rev. Henry Sharpe and the Rev. J. A. Bevan.

Friedenheim itself consists of a villa residence of which the best use has been made, and the wards are on the whole bright and airy. It has a large garden at the back, and certain of the wards have verandahs for the use of the patients. There is also a large conservatory or winter garden for rainy days. Each ward has a sitting-room adjacent to it, where those patients who are able to get up can sit and take their meals. Miss Davidson has also taken care to provide that most necessary of all things for a Home for the Dying—a well-constructed mortuary, which for simplicity and suitability leaves little to desire. Friedenheim is rather hampered for want of funds, and an additional £300 a year in income, and an immediate gift of £2,000 in donations, would enable the committee to introduce many improvements which would add to the efficiency of the buildings in more than one direction.

THE HOSTEL OF GOD—FREE HOME FOR THE DYING.

The work of this institution has been carried on for some time in an ordinary terrace house in Clapham, in every way unsuitable to the purpose, and under very considerable difficulties. The home is intended solely for the care of the dying, and is not for the cure of disease; and therefore is not, in the ordinary sense of the word, a hospital. But although the cure of disease is not undertaken, it is obvious that a certain amount of treatment must form an essential part of the work; and to this end, and also for the hygienic welfare of the patients and the staff, the building must necessarily take the form, to some extent, of a hospital. The majority of the cases admitted are either cancer patients or are suffering from the class of lung diseases included under the general term of consumption.

It is proposed, when a proper site and funds are forthcoming, to build a suitable institution, for which plans are already prepared, which are likely to be entirely re-arranged. The plans as they stand provide for twenty-four patients in wards of different sizes, arranged on two floors. The large wards of six beds are intended for patients who, from the nature of their disease can without disadvantage, be associated together; wards for two beds would be used for patients for whom quiet was more desirable; while one-bed wards would be provided for cases *in extremis*, or who could not without discomfort be placed in wards with other patients. In the large wards the allowance of cubic space per bed is 1,500 feet, the space in the smaller wards being proportionately greater.

The south wing will be the chapel. Access to this is provided for the patients on both ground and first floors; in the latter case the corridor communicating with a gallery at the west end of the chapel, this arrangement being specially devised for the benefit of patients the nature of whose disease renders the ascent and descent of stairs undesirable. An open cloister would also provide access to the chapel from the sisters' wing without passing along the patients' corridor. The south transept of the chapel will be arranged to be used as a mortuary.

The Home will be governed by the East Grinstead sisters, assisted by trained nurses; the residence of both sisters and nurses being contained in the north wing. The kitchen offices and servants' quarters will be in the west wing, over the first floor of wards.

The building, a view of which appeared in this year's Royal Academy exhibition, has been designed by Messrs. Young and Hall.

